# Investigating the Efficacy of Various Natural Products in Raw Form against Multidrug-Resistant Bacteria

**DOI:** 10.2174/0118715265320631240826073359

**Published:** 2024-09-20

**Authors:** Hamad H. Alanazi, Hussain Ali G. Aldughmani, Bi Bi Zianab Mazhari

**Affiliations:** 1 Department of Clinical Laboratory Science, College of Applied Medical Sciences-Qurayyat, Jouf University, Al Jouf, Saudi Arabia;; 2 Department of Microbiology, Quryyat Hospital, Quryyat Regional Laboratory, Al Qurayyat, Saudi Arabia

**Keywords:** Phytochemicals, antibiotic resistance, antimicrobial drugs, natural products, allium sativum, garlic, antibiotics

## Abstract

**Background:**

The alarming increase in antibiotic resistance urges alternative and efficacious antimicrobial solutions. Historically, medicinal plants have been used for therapeutic purposes, such as relieving pain and healing wounds. The evaluation of the natural therapeutic effects of medicinal plants in a manner that resembles how humans typically consume them is lacking. In this study, many medicinal plants known to have some antimicrobial effects, including Frankincense, Garlic, Myrrh, and Ginger, were evaluated for their direct antibacterial activity in raw form.

**Materials and Methods:**

The direct antimicrobial activity of medicinal plants was evaluated against a variety of Gram-positive and Gram-negative bacterial strains, such as *Staphylococcus aureus* (*S. aureus.*), *Acinetobacter baumannii* and *Klebsiella pneumoniae* using agar well diffusion method and turbidity measurements in suspension culture.

**Results:**

Out of all the tested medicinal plants, only raw garlic (*Allium sativum*) powder, when dissolved in water or vinegar, offered a straightforward antibacterial activity. A combination of garlic extract and vinegar increased antibacterial activity. Aqueous garlic extracts displayed robust antimicrobial activity against many resistant bacteria. Other medicinal plants used in this study had absent or minimal antibacterial effects.

**Conclusion:**

Only garlic in its raw form was effective against antibiotic-resistant bacteria. The increase in the antibacterial activity of garlic when combined with vinegar suggests synergistic activity of garlic. The straightforward antibacterial action of raw garlic may be strategically harnessed to combat the continuous challenge of increasing antibiotic resistance. This work promotes additional testing of more natural products (in raw form) and assesses their therapeutic effects clinically.

## 1 INTRODUCTION

In the face of increasing antimicrobial resistance and the limited effectiveness of conventional antimicrobial agents, there is a growing interest in exploring alternative approaches to combat microbes. Phytochemicals, naturally occurring compounds found in plants, have emerged as potential candidates for antimicrobial defense [1]. These bioactive compounds possess diverse therapeutic properties and have shown promise in enhancing the body's defenses against microbial threats [2]. Our work explores the antibacterial effects of common phytochemicals, shedding light on their potential applications in combating bacterial diseases. Phytochemicals have long been recognized for their crucial roles in plants, contributing to their defense mechanisms against pathogens and environmental stressors [3]. As humans consume plant-based foods and herbal remedies, they can benefit from the bioactive compounds present in these plants. Over the years, scientific research has unraveled the multifaceted therapeutic potential of phytochemicals, highlighting their antimicrobial activity against bacteria, viruses, fungi, and other microorganisms [4]. One of the significant advantages of phytochemicals in antimicrobial defense is their broad-spectrum activity, meaning they can target multiple types of pathogens at the same time [5]. This versatility is essential in combating infectious diseases that often involve mixed infections or evolved drug-resistant strains. By targeting various stages of microbial growth and replication, phytochemicals offer a multifaceted approach to tackling microbial infections.

Moreover, many phytochemicals exhibit complementary properties that support the body's innate immune system. For instance, certain compounds possess potent antioxidant and anti-inflammatory effects, which can help mitigate the damaging effects of microbial-induced inflammation and oxidative stress [6, 7]. By modulating the immune response, phytochemicals contribute to a more effective defense against microbial invaders. The therapeutic effects of phytochemicals in antimicrobial defense have been demonstrated through many *in vitro* and *in vivo* studies [8]. These investigations have highlighted the ability of phytochemicals to inhibit microbial growth, disrupt biofilm formation, interfere with microbial gene expression, and enhance the activity of immune cells.

Additionally, some phytochemicals have synergistic effects with conventional antimicrobial agents, potentiating their efficacy and minimizing the development of resistance [9]. While the potential of phytochemicals in antimicrobial defense is promising, further research is essential to harness their full therapeutic potential. Factors, such as optimal dosage, bioavailability, and stability require further investigation to ensure their effective use in clinical settings. Additionally, more comprehensive clinical trials are warranted to evaluate the safety and efficacy of phytochemical-based treatments in various infectious diseases.

Exploring the therapeutic effects of common phytochemicals in antimicrobial defense offers a promising avenue for combating microbial infections. The multifaceted properties of phytochemicals, ranging from direct antimicrobial activity to immune modulation, make them attractive candidates for developing alternative antimicrobial therapies. By investigating their mechanisms of action deeper and conducting rigorous clinical studies, we can unlock the full potential of phytochemicals and pave the way for novel strategies in antimicrobial defense. In this regard, we aimed to test the antibacterial activity of various natural products, including garlic, myrrh, ginger, chamomile, and others, against common gram-positive and gram-negative bacteria.

One of the most important medicinal plants used for therapeutic purposes is garlic [10, 11]. Garlic has several antimicrobial effects, such as bacterial, viral, and fungal growth inhibition [12, 13]. The antibacterial effects of garlic were described in 1858 by Louis Pasture [14]. Moreover, studies have shown that garlic has anticancer effects and can reduce high blood pressure and glucose levels [15]. Many of the therapeutic effects of garlic are attributed to allicin, the bioactive compound found in garlic [14]. The methods commonly used to evaluate antibacterial activity of garlic typically involves alcohol [16]. In this study, the antibacterial effect of garlic is measured using garlic dissolved in distilled water to mimic how people typically consume it.

To obtain optimal insights into how these natural products affect the human body, we prepared them in this study the same way they are typically prepared and consumed by individuals. Using the agar well diffusion method, we tested the antimicrobial activity of numerous natural products and determined their antibiotic effects against common and resistant bacterial strains.

## 2 MATERIALS AND METHODS

### 2.1 Plant Material

All plant substances used for antibiotic evaluation were obtained commercially, including black seed, garlic paste, frankincense, wormwood, barley grain, turmeric, garlic powder, onion powder, garlic oil, myrrh, and ginger. All plant materials except garlic oil were soaked in warm distilled water according to the indicated concentrations to prepare the water extract solutions. The concentration used in the experiments was the weight of extract per ml. The extract solutions were incubated overnight at room temperature. The extract solutions were centrifuged at 3000 rpm for 10 min using a 10mL tube. The volume dispensed in each agar well was 130 ul for all experiments.

### 2.2 Antibacterial Activity and Media

The diameter of the inhibition zone was measured in mm to assess the antibacterial activity of each extract. Amoxicillin was used as a positive control, while water served as a negative control. The agar well diffusion method using Mueller-Hinton agar media was used to measure the antibacterial activity of all extracts. To monitor the efficacy of antibacterial activity over seven days, Luria Broth (LB) media was used. A 37.5 mg/mL concentration of 7 mL LB media + 1 mL extract solution was used. After the first day, 20 ul of bacterial solution was added to all tubes except negative control.

### 2.3 Well Diffusion Method

Using a sterile cotton swab, bacterial suspensions with a McFarland standard of 0.5 were applied onto Mueller-Hinton agar plates. After allowing the plates to air dry for 15 minutes, wells with an 8-millimeter diameter were created using a cork borer. Each well was then filled with 130 microliters. Amoxicillin was filled in well on the plate as a reference antibiotic against bacterial species to serve as a positive control. Following an incubation period of 24 hours, the diameter of the inhibition zone surrounding each well was measured, and photographic documentation of the plates was performed.

### 2.4 Bacterial Strains

*Staphylococcus aureus* (ATCC 29213), *Escherichia coli* (ATCC 25922), and multidrug-resistant strains of vancomycin-resistant enterococcus (VRE), methicillin-resistant *Staphylococcus aureus* (MRSA), *Acinetobacter baumannii* (AbA), and carbapenem-resistant Enterobacteriaceae (CRE) were used for testing the antibiotic activity of products. The bacterial isolates used in the study were obtained from samples provided by patients at the hospital.

### 2.5 Statistical Analysis

Statistical analyses were performed using a 2-tailed unpaired t-test (2 groups). *p* < 0.05 was considered statistically significant. Data are expressed as the mean ± SD in the manuscript.

## 3 RESULTS

**Fig. (1):**
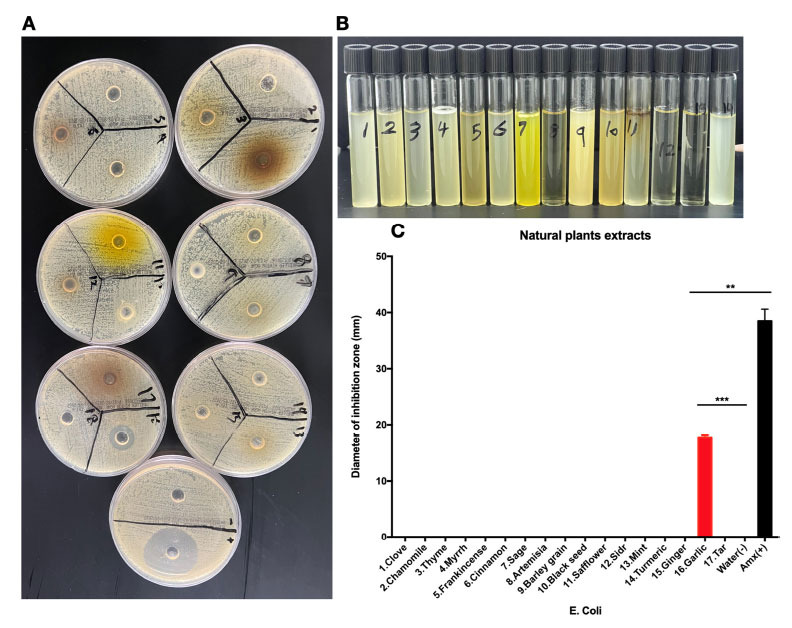
**Antibiotic effects of common natural products.** (**A**) Several products were tested for antibacterial effects using the Well Diffusion Method using a concentration of (13 mg/well). (**B**) Many other products with the following order: 1-Aleppo stone, 2-Black seed, 3-Garlic paste, 4-The Frankincense, 5-Wormwood, 6-Barley grain, 7-Tumeric, 8-Garlic powder, 9-Myrrh, 10-Ginger, 11-Tar, 12-Amx, 13-Negative control, and 14-Positive control were tested for their antibacterial effects in broth media (LB) using a concentration of (31 mg/ml). (**C**) In reference to (**A**), each substance's diameter of the zone of inhibition (mm) was measured.

We wanted to explore the antibacterial activity of various natural products that were believed to have antibacterial effects using the agar well diffusion method. The range of garlic concentration that must be used to observe the antibacterial effects is (15-60 mg/ml) [17]. To mimic the way people usually consume natural products, we prepared aqueous extracts of natural products by dissolving extracts in water. Most compounds tested were ineffective in killing the bacteria (*E. coli*) at low concentrations (Fig. **1A**). However, sample 16, which contained garlic solution, showed antibacterial activity by a clear zone of inhibition (Fig. **1A**). Using a different method, we tested the efficacy of more compounds in addition to garlic, and consistent with Fig. (**1A**), we found that only garlic at a lower concentration stopped bacterial growth (Fig. **1B**). We measured the diameter of the inhibition zone and found that it was ~20 mm, almost half of the diameter for the positive control Amoxicillin (Fig. **1C**). These results show that among many beneficial natural products believed to have antibiotic activity, only garlic at a low concentration of 13 mg/well (Fig. **1A**) and 31 mg/mL (Fig. **1B**) could inhibit bacterial growth. This antibacterial inhibition was dose-dependent, as shown in Supplementary Fig. (**S1**).

**Fig. (2):**
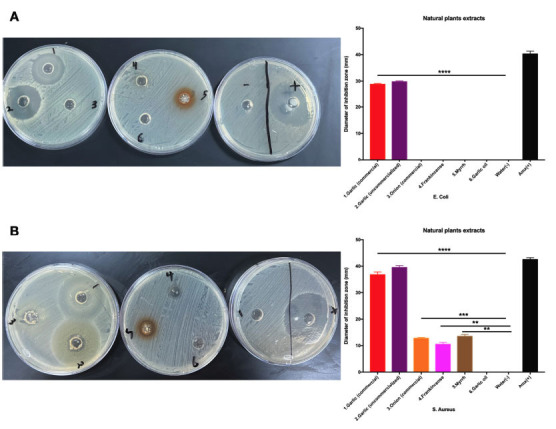
**Antibiotic effects of garlic against gram-negative and gram-positive bacteria.** (**A**) The antibacterial activity of 1. Garlic (commercial), 2. Garlic (local), 3.Onion (commercial), 4. Frankincense, 5.Myrrh, 6. Garlic Oil and negative (water) and positive controls (Amoxicillin) were tested in Mueller-Hinton agar cultured with *E. coli* using a concentration of (39 mg/well). (**B**) Similar to (**A**), except Mueller-Hinton agar was cultured with *S. aureus.*

Our next focus was to examine garlic's antibiotic effects, not only against gram-negative *E. coli* but also against a common representative of gram-positive bacteria (*S. aureus.*). The results were striking, as garlic alone, compared to other natural compounds, was able to effectively inhibit bacterial growth in both Figs. (**2A** and **B**). Furthermore, both commercialized and uncommercialized garlic demonstrated similar antibacterial activities against *E. coli and S. aureus* (Fig. **2A** and **B**). Of note other natural products, like onion, frankincense, and myrrh, showed only slight antibacterial activity against *S. aureus* (Fig. **2B**). This highlights the need for further research to fully understand the antibacterial properties of these natural products. In summary, our study reveals that garlic, at a concentration of 39 mg/well, exhibits universal antibacterial effects against both gram-negative and gram-positive bacteria, while the antibacterial activity of other natural products was either absent against *E. coli* or very low against *S. aureus.*

**Fig. (3):**
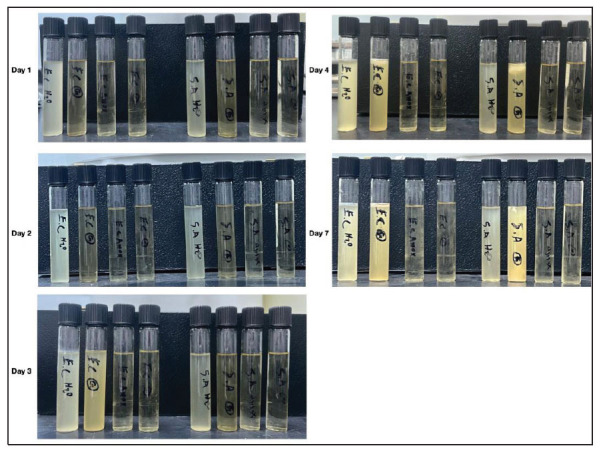
**Monitoring gram-negative and gram-positive bacterial growth in LB media in the presence of garlic**. Bacterial suspension of *E. coli* and *S. aureus.* in LB media containing H_2_O, Garlic (37.5 mg/mL), Amoxicillin (positive), and LB media without bacterial growth (negative). Tubes were maintained in a 37°C incubator for the indicated days. Each day, samples were vortexed and returned to the incubator.

To test how long the antibacterial activity of garlic lasted compared to a positive control (Amoxicillin), we grew bacteria in the presence of garlic. A concentration of 37.5 mg/mL of garlic inhibited the growth of *E. coli* and *S. aureus.* after one day of bacterial inoculation in LB media (Fig. **3**). On day 2, garlic inhibited bacterial growth (*E. coli* and *S. aureus.* bacteria), similar to Amoxicillin. On day 3, the garlic activity started to fade away, which allowed the growth of *E. coli*. However, *S. aureus.* remained the same, suggesting stronger resistance for *E. coli* against natural antibiotics (Fig. **3**). On days 4 and 7, garlic activity was lost for all bacterial strains grown in LB media, thus allowing bacteria to revive (Fig. **3**). However, no bacterial growth was noticed in the Amoxicillin sample, suggesting that synthesized antibiotics completely eliminated bacterial organisms (Fig. **3**). These results suggest that, unlike modern antibiotics, natural antibiotic activity can be transient.

**Fig. (4):**
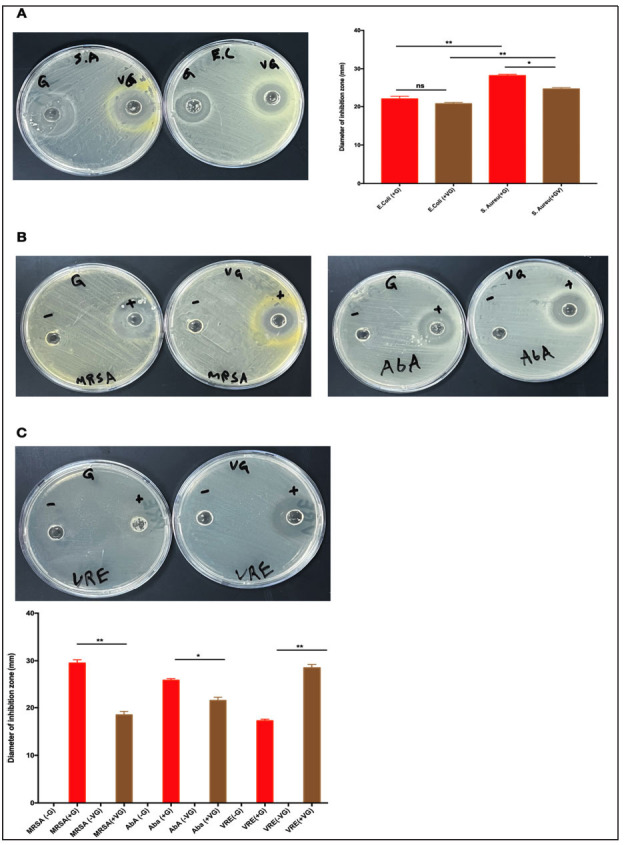
**Testing the antibiotic effects of garlic against several multi-drug resistant bacteria.** (**A**) Antibacterial activity of garlic alone and garlic+vinegar against *S. aureus.* and *E. coli* culture on Mueller-Hinton agar using a concentration of (32.5 mg/well). (**B, C**) Antibacterial activity of garlic alone and garlic+vinegar against resistant bacteria (MRSA, AbA, VRE) using a concentration of (32.5mg/well).

**Fig. (5):**
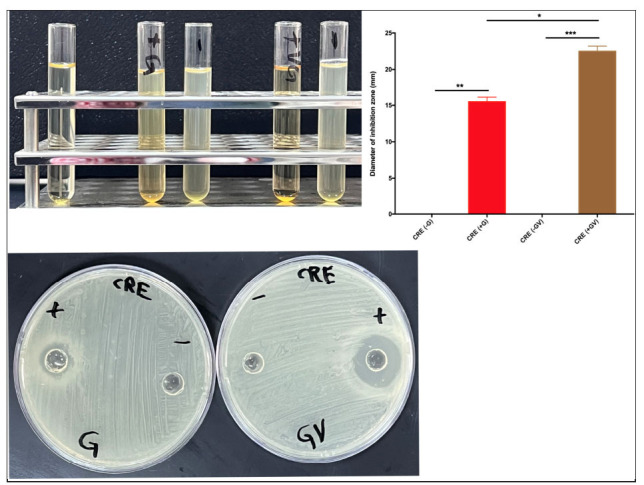
**Testing garlic’s antibacterial effects against *carbapenem-resistant Enterobacteriaceae* (CRE), mainly *Klebsiella pneumoniae*.** Antibacterial activity of garlic alone and garlic+vinegar (32.5 mg/well) against CRE culture on Mueller-Hinton agar.

To test whether garlic can synergistically work with other natural substances, we tested the efficacy of garlic in combination with vinegar, against *E. coli* and *S. aureu*s. The combination of garlic and vinegar had antibacterial activity against several resistant bacteria, not only *E. coli* and *S. aureus* (Supplementary Figs. **S2**-**S4**). We then compared the antibacterial activity of garlic alone and garlic+vinegar against four resistant bacteria. As expected, garlic and garlic+vinegar had antibacterial activity against *E. coli* and *S. aureus.* (Fig. **4A**). The antibacterial activity of garlic was the highest against MRSA, reflected by the diameter of the inhibition zone 29.5 mm (Figs. **4B** and **C**). The inhibition zone of garlic against AbA was 25.75 mm, and against VRE was 17.25 mm (Figs. **4B** and **C**). Inversely, the inhibition zones of garlic+vinegar against VRE, AbA, and MRSA were 28.5, 21.5, and 18.5 mm, respectively (Figs. **4B** and **C**). We also tested the antibacterial activity of garlic and garlic+vinegar against (CRE), mainly Klebsiella pneumoniae. The zone of inhibition for garlic+vinegar was 22 mm, while for garlic alone, it was 15 mm (Fig. **5**). Together, these results suggest that natural antibiotics can work independently or synergistically to inhibit bacterial growth of resistant bacteria.

## 4 DISCUSSION

The rate of bacterial resistance is increasing over time due to the overuse of antibiotics [18, 19]. The findings of our study emphasize the therapeutic role of garlic solution as an alternative antimicrobial treatment for resistant bacteria. Many studies showed garlic has antibiotic activity against common bacteria [20]. In this study, we investigated various common products known for their antimicrobial properties, including Aleppo stone, Black seed, Garlic paste, Frankincense, Wormwood, Barley grain, Tumeric, Garlic powder, Myrrh, Ginger, Tar, and onion. Our study showed that only raw garlic powder, when dissolved in water, exhibited a potent and straightforward antibacterial activity compared to other products.

Garlic extract solution, without any purification methods, demonstrated robust antimicrobial activity against a diverse range of bacteria, including both Gram-positive and Gram-negative strains, such as (VRE), (MRSA), (AbA), and *Klebsiella pneumonia.* These data suggest the broad-spectrum activity of garlic against multidrug-resistant bacterial infections. The inhibition of bacterial growth by garlic solution persisted for 2-3 days compared to synthetic Amoxicillin, which completely seized further bacterial growth. The persistent inhibition of bacterial growth (Supplementary Fig. **S5**) may offer an effective strategy for combating bacterial infections, especially those resistant to synthesized conventional antibiotics.

This study highlights the importance of exploring natural products in their original and unaltered state for their therapeutic potential. Garlic, in its original and unaltered state, is consumed by people daily, although it shows antibacterial effects similar to conventional antibiotics. This raises some concerns, given that some medications can not be prescribed together with antibiotics [21]. Also, consuming garlic may alter the microenvironment of normal flora, which is considered part of the immune system [22].

Although many studies have emphasized the antibiotic role of natural products, like garlic, it is essential to perform *in vitro* and *in vivo* studies to further demonstrate the therapeutic effects of these products in clinical settings. Moreover, it would be appealing to investigate other natural products that may uncover additional targets for defending against antibiotic-resistant bacteria. Furthermore, it would be intriguing to test the efficacy of garlic in its unaltered state against other resistant strains of bacteria, viruses, and fungi.

## CONCLUSION

In conclusion, this study highlights the potential of garlic as an alternative and promising strategy against increasing antibiotic resistance. In addition, this study opens the door to exploring the therapeutic benefits of other natural products in their unaltered state. Continuous exposure to natural antibiotics contributes beneficially to overall immunity against bacteria; however, it may have negative implications, such as altering the microbiota homeostasis.

## Data Availability

The data and supportive information are available within the article.

## LIST OF ABBREVIATIONS

AbA: Acinetobacter Baumannii
CRE: Carbapenem-resistant Enterobacteriaceae
LB: Luria Broth
MRSA: Methicillin-resistant *Staphylococcus aureus*
VRE: Vancomycin-resistant Enterococcus
