# Supplementary material for: Investigating the Efficacy of Various Natural Products in Raw Form against Multidrug-Resistant Bacteria
Source: Infect Disord Drug Targets. 2024 Sep 20;25(3):E18715265320631. doi: 10.2174/0118715265320631240826073359 (PMC11851147; doi:10.2174/0118715265320631240826073359)
Supplement: Supplementary file 1 [file IDDT-25-3-E18715265320631_SD1.pdf]

Supplementary Material

Investigating the Efficacy of Various Natural Products in Raw Form against Multidrug-Resistant Bacteria

Hamad H. Alanazi[1,*], Hussain Ali G Aldughmani[2] and Bi Bi Zianab Mazhari[1]

[1]Department of Clinical Laboratory Science, College of Applied Medical Sciences-Qurayyat, Jouf University, Al Jouf, Saudi Arabia; [2]Department of Microbiology, Quryyat Hospital, Quryyat Regional Laboratory

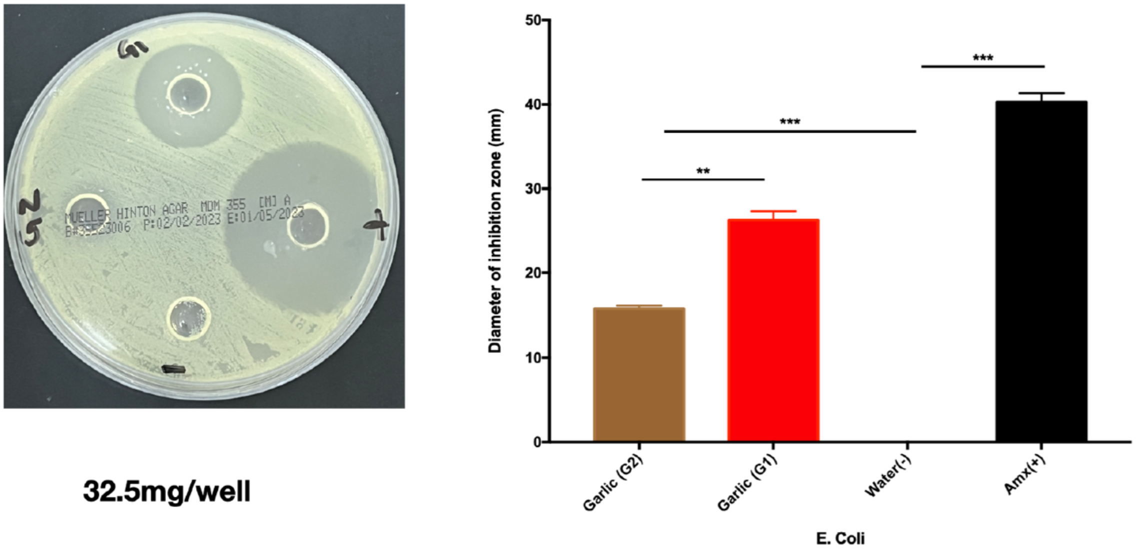

Fig. (S1). Antibiotic effects of garlic. Different dilutions of garlic were used to measure antibacterial effects. G1=32.5mg/ml, G2=16.25mg/ml.

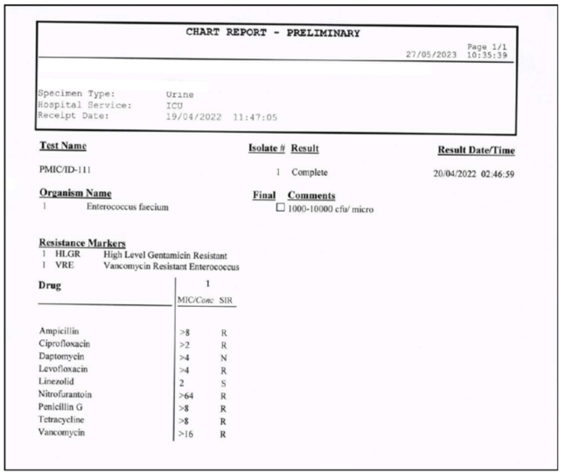

CHART REPORT - PRELIMINARY

Page 1/1
27/05/2023 10:35:39

Specimen Type: Urine
Hospital Service: ICU
Receipt Date: 19/04/2022 11:47:05

Test Name	Isolate #	Result	Result Date/Time
PMIC/ID-111	1	Complete	20/04/2022 02:46:59

Organism Name		Final	Comments
1	Enterococcus faecium	☐	1000-10000 cfu/ micro

Resistance Markers
1 HLGR High Level Gentamicin Resistant
1 VRE Vancomycin Resistant Enterococcus

Drug	1 MIC/Conc	SIR
Ampicillin	>8	R
Ciprofloxacin	>2	R
Daptomycin	>4	N
Levofloxacin	>4	R
Linezolid	2	S
Nitrofurantoin	>64	R
Penicillin G	>8	R
Tetracycline	>8	R
Vancomycin	>16	R

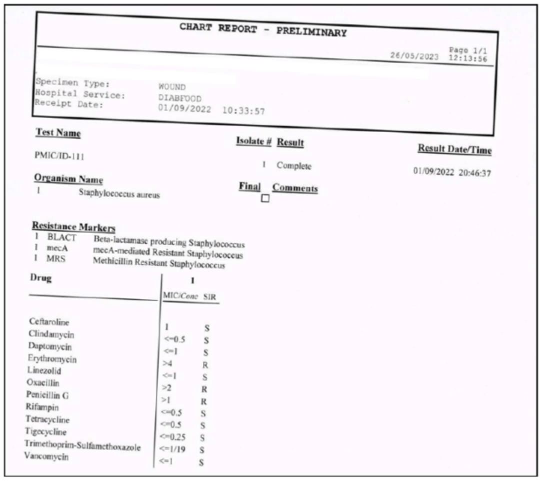

CHART REPORT - PRELIMINARY

Page 1/1
26/05/2023 12:13:56

Specimen Type: WOUND
Hospital Service: DIABFOOD
Receipt Date: 01/09/2022 10:33:57

Test Name	Isolate #	Result	Result Date/Time
PMIC/ID-111	1	Complete	01/09/2022 20:46:37

Organism Name		Final	Comments
1	Staphylococcus aureus	☐	

Resistance Markers
1 BLACT Beta-lactamase producing Staphylococcus
1 mecA mecA-mediated Resistant Staphylococcus
1 MRS Methicillin Resistant Staphylococcus

Drug	1 MIC/Conc	SIR
Ceftaroline	1	S
Clindamycin	<=0.5	S
Daptomycin	<=1	S
Erythromycin	>4	R
Linezolid	<=1	S
Oxacillin	>2	R
Penicillin G	>1	R
Rifampin	<=0.5	S
Tetracycline	<=0.5	S
Tigecycline	<=0.25	S
Trimethoprim-Sulfamethoxazole	<=1/19	S
Vancomycin	<=1	S

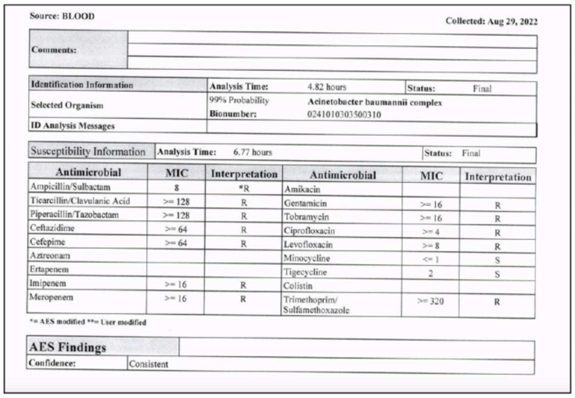

Source: BLOOD
Collected: Aug 29, 2022

Comments:

Identification Information	Analysis Time:	4.82 hours	Status: Final
Selected Organism	99% Probability<br>Bionumber:	**Acinetobacter baumannii complex**<br>0241010303500310	
ID Analysis Messages			

Susceptibility Information — Analysis Time: 6.77 hours — Status: Final

Antimicrobial	MIC	Interpretation	Antimicrobial	MIC	Interpretation
Ampicillin/Sulbactam	8	*R	Amikacin		
Ticarcillin/Clavulanic Acid	>= 128	R	Gentamicin	>= 16	R
Piperacillin/Tazobactam	>= 128	R	Tobramycin	>= 16	R
Ceftazidime	>= 64	R	Ciprofloxacin	>= 4	R
Cefepime	>= 64	R	Levofloxacin	>= 8	R
Aztreonam			Minocycline	<= 1	S
Ertapenem			Tigecycline	2	S
Imipenem	>= 16	R	Colistin		
Meropenem	>= 16	R	Trimethoprim/ Sulfamethoxazole	>= 320	R

*= AES modified **= User modified

AES Findings	
Confidence:	Consistent

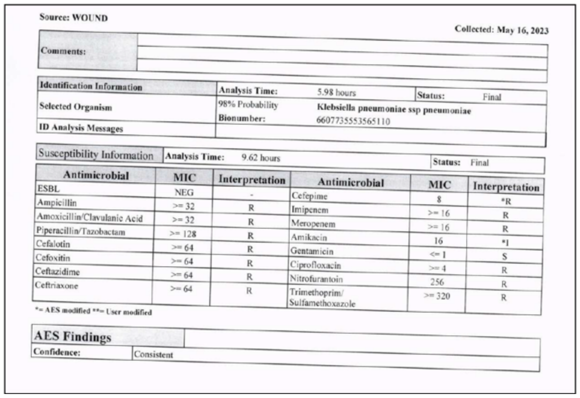

Source: WOUND
Collected: May 16, 2023

Comments:

Identification Information	Analysis Time:	5.98 hours	Status: Final
Selected Organism	98% Probability<br>Bionumber:	**Klebsiella pneumoniae ssp pneumoniae**<br>6607735553565110	
ID Analysis Messages			

Susceptibility Information — Analysis Time: 9.62 hours — Status: Final

Antimicrobial	MIC	Interpretation	Antimicrobial	MIC	Interpretation
ESBL	NEG	-	Cefepime	8	*R
Ampicillin	>= 32	R	Imipenem	>= 16	R
Amoxicillin/Clavulanic Acid	>= 32	R	Meropenem	>= 16	R
Piperacillin/Tazobactam	>= 128	R	Amikacin	16	*I
Cefalotin	>= 64	R	Gentamicin	<= 1	S
Cefoxitin	>= 64	R	Ciprofloxacin	>= 4	R
Ceftazidime	>= 64	R	Nitrofurantoin	256	R
Ceftriaxone	>= 64	R	Trimethoprim/ Sulfamethoxazole	>= 320	R

*= AES modified **= User modified

AES Findings	
Confidence:	Consistent

Fig. (S2). Multi-drug resistant bacterial isolates. Report of bacteria isolated from patient samples (origin of the sample, type of antibiotics tested, S=Sensitive, R=Resistant).

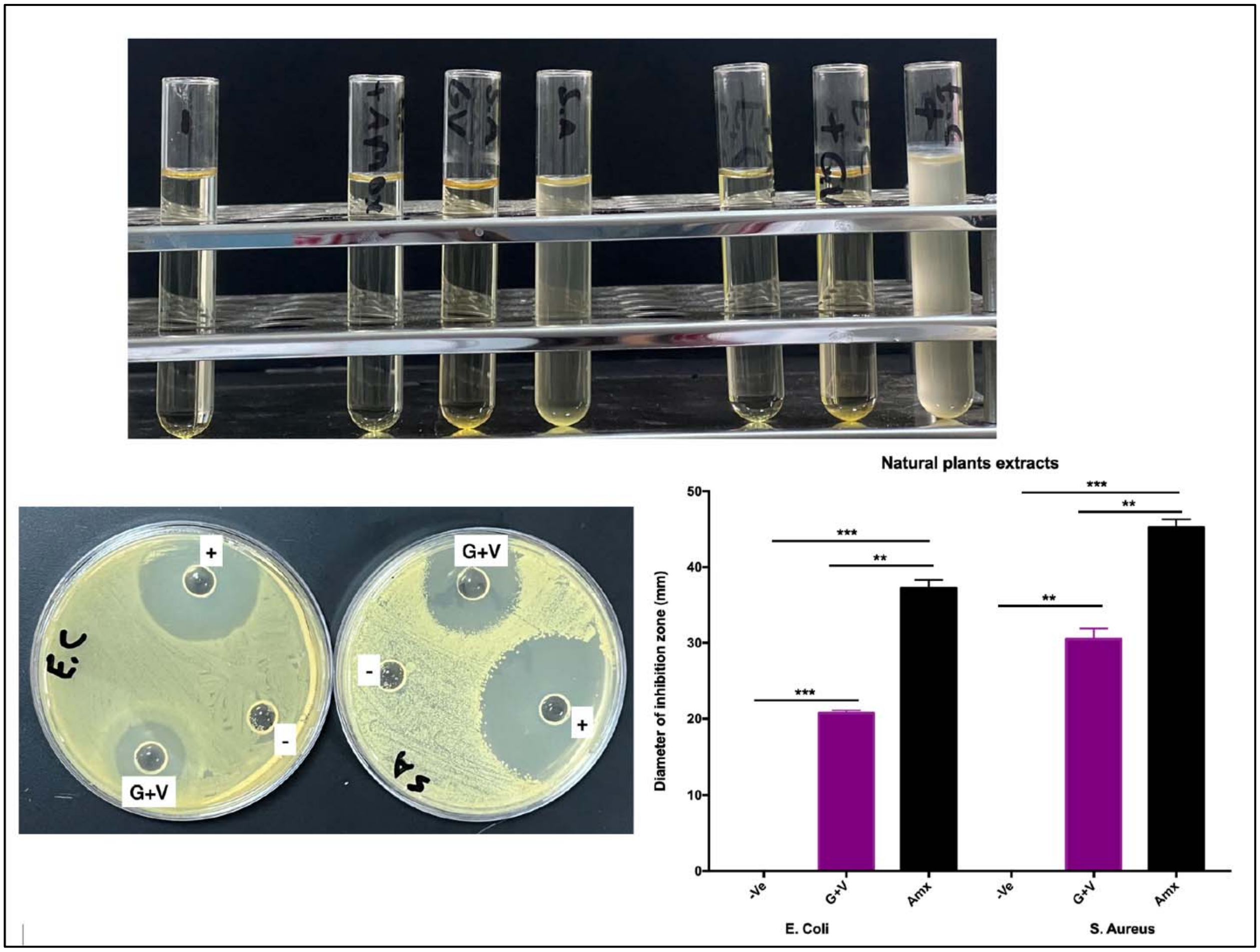

Fig. (S3). Antibacterial combined effects of garlic and vinegar against bacteria. Bacterial growth in the presence of garlic+vinegar. *S. aureus* is gram positive and E. Coli is gram negative.
.

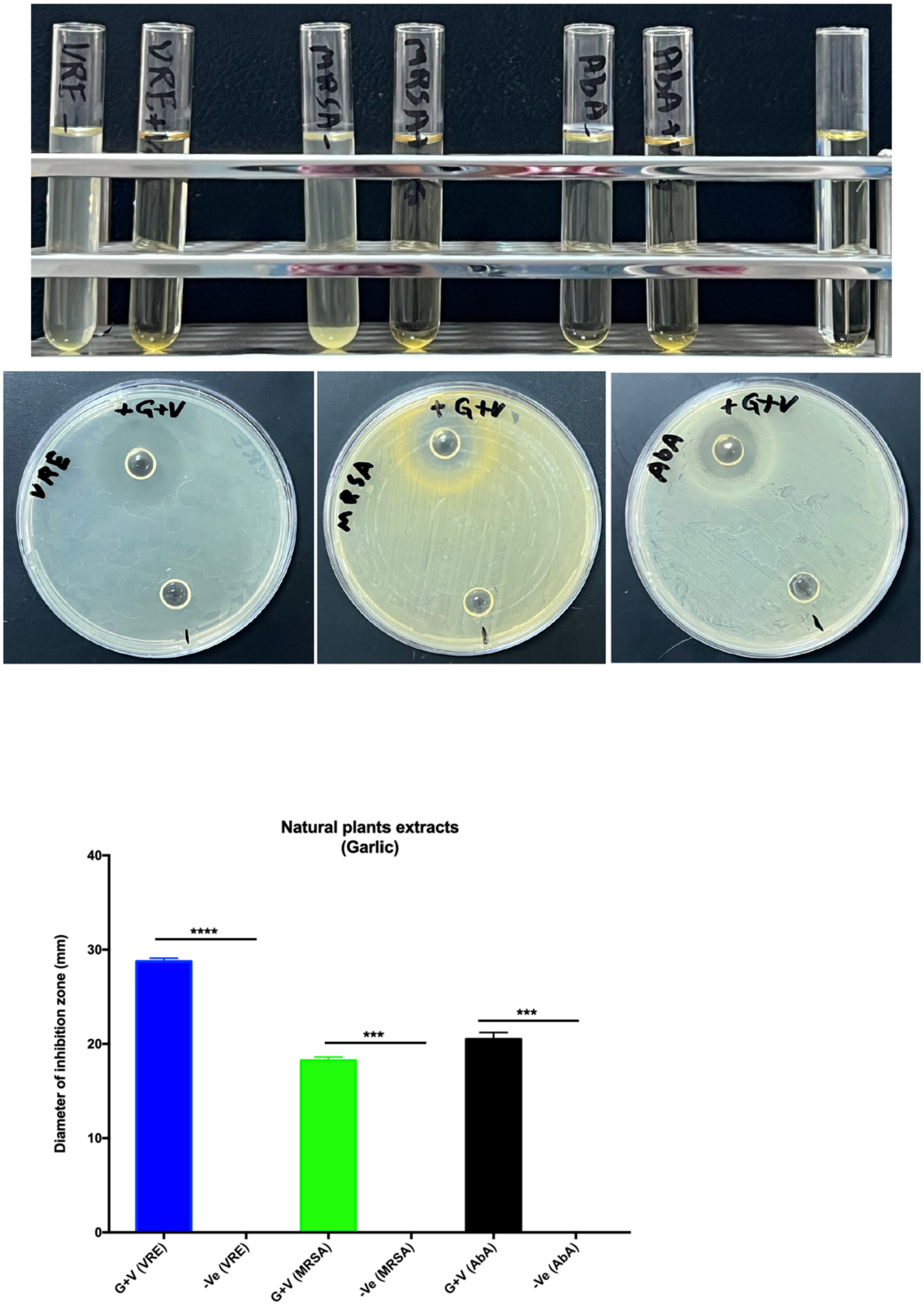

Fig. (S4). Antibacterial combined effects of garlic and vinegar against antibiotic-resistant bacteria. Bacterial growth in the presence of garlic+vinegar. The effect of garlic and vinegar was tested against VRE, MRSA, and AbA.

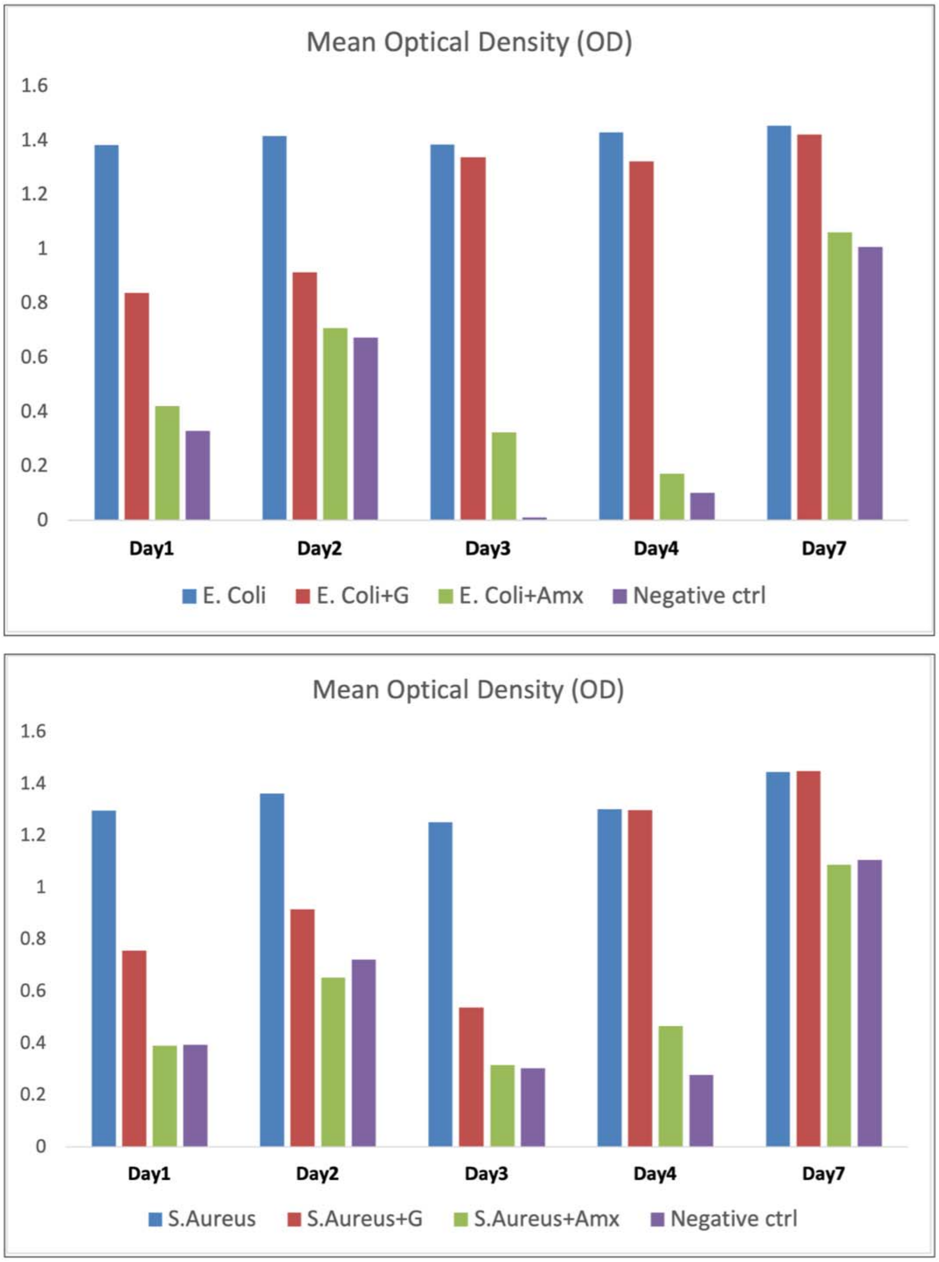

Fig. (S5). Optical density (OD) at 600 am. OD of bacterial growth measurements at indicated days concerning Fig. (3).
